# Modelling Combined Intravenous Thrombolysis and Mechanical Thrombectomy in Acute Ischaemic Stroke: Understanding the Relationship between Stent Retriever Configuration and Clot Lysis Mechanisms

**DOI:** 10.3390/life11111271

**Published:** 2021-11-20

**Authors:** Emily Louise Manchester, Dylan Roi, Boram Gu, Xiao Yun Xu, Kyriakos Lobotesis

**Affiliations:** 1 Department of Chemical Engineering, Imperial College London, South Kensington Campus, London SW7 2AZ, UK; elm17@ic.ac.uk (E.L.M.); boram.gu@chonnam.ac.kr (B.G.); 2 Imaging Department, Charing Cross Hospital, Imperial College Healthcare NHS Trust, London W6 8RF, UK; dylan.roi@nhs.net; 3 School of Chemical Engineering, Chonnam National University, Gwangju 61186, Korea

**Keywords:** stroke, intravenous thrombolysis, mechanical thrombectomy, stent retriever, numerical model, computational fluid dynamics, blood flow, carotid artery, cerebral artery

## Abstract

**Background**: Combined intravenous thrombolysis and mechanical thrombectomy (IVT-MT) is a common treatment in acute ischaemic stroke, however the interaction between IVT and MT from a physiological standpoint is poorly understood. In this pilot study, we conduct numerical simulations of combined IVT-MT with various idealised stent retriever configurations to evaluate performance in terms of complete recanalisation times and lysis patterns. **Methods**: A 3D patient-specific geometry of a terminal internal carotid artery with anterior and middle cerebral arteries is reconstructed, and a thrombus is artificially implanted in the MCA branch. Various idealised stent retriever configurations are implemented by varying stent diameter and stent placement, and a configuration without a stent retriever provides a baseline for comparison. A previously validated multi-level model of thrombolysis is used, which incorporates blood flow, drug transport, and fibrinolytic reactions within a fibrin thrombus. **Results**: Fastest total recanalisation was achieved in the thrombus without a stent retriever, with lysis times increasing with stent retriever diameter. Two mechanisms of clot lysis were established: axial and radial permeation. Axial permeation from the clot front was the primary mechanism of lysis in all configurations, as it facilitated increased protein binding with fibrin fibres. Introducing a stent retriever channel allowed for radial permeation, which occurred at the fluid-thrombus interface, although lysis was much slower in the radial direction because of weaker secondary velocities. **Conclusions**: Numerical models can be used to better understand the complex physiological relationship between IVT and MT. Two different mechanisms of lysis were established, providing a basis towards improving the efficacy of combined treatments.

## 1. Introduction

Acute ischaemic stroke (AIS) is the second most common cause of mortality worldwide, and is associated with significant morbidity in survivors [1]. Thrombolytic therapy, and, more recently, mechanical thrombectomy have improved outcomes for patients. A large proportion of patients eligible for mechanical thrombectomy (MT) are also candidates for intravenous thrombolysis (IVT). Meta-analyses, including the larger thrombectomy randomised trials [2] and another based on studies during the last 3 years [3], found that compared to MT alone, IVT prior to MT led to better clinical outcomes, lower mortality, and higher rates of successful recanalisation. Contrary to this, a meta-analysis of randomised clinical trials (MR CLEAN-NO IV [4], DIRECT-MT [5], DEVT [6], and SKIP [7]) found that MT alone is non-inferior to IVT-MT based on functional independence at 90 days [8]. Thrombolysis can result in intracranial haemorrhage (ICH), with associated morbidity and mortality, however, there was no significant difference in ICH rate between thrombectomy alone and combination therapy [2,3,5]. Based on recent meta-analyses, uncertainty remains on direct MT versus combined IVT-MT treatment superiority.

We previously developed a multi-level numerical model of thrombolysis which incorporates blood flow, drug transport, and fibrinolytic reactions within a fibrin thrombus [9,10]. The model also provides a physiological inlet boundary condition which includes pharmacokinetic and pharmacodynamic (PKPD) effects on fibrinolytic proteins in plasma following intravenous administration of tissue plasminogen activator (tPA). The model was applied in patient-specific settings with middle cerebral artery (MCA) thrombi to evaluate the effects of drug dosage and thrombus density on lysis completion times and risk of ICH [9]. Results showed that for all drug dosages, tPA penetration through dense clots were much slower than penetration through coarser clots, resulting in slower lysis times. The model was also used to evaluate the influence of thrombus size and location (M1 and M2 occlusions) on lysis completion times and haemodynamic characteristics [10]. Findings supported clinical observations that recanalisation success and lysis times were dependent on clot size. More specifically, lysis rates were dependent on arterial curvature and thrombus location, where accumulation of tPA at the clot front accelerated lysis rates.

Though routinely performed in tandem, the interaction between IVT and MT has not previously been modelled, and there is a lack of mechanistic understanding of their combined effect on treatment efficacy. Using our existing thrombolysis model in a patient-specific geometry of the internal carotid artery, and the anterior and middle cerebral arteries, we conduct a pilot numerical study into combined IVT-MT treatment. Considering the extremely high computational demand for simulations of this kind, various idealised stent retriever configurations are adopted in an MCA thrombus. Combined treatment performance is evaluated in terms of complete recanalisation times and lysis patterns, and recommendations for future work in this emerging field are provided.

## 2. Materials and Methods

### 2.1. Arterial Geometry and Clot Composition

**Figure 1:**
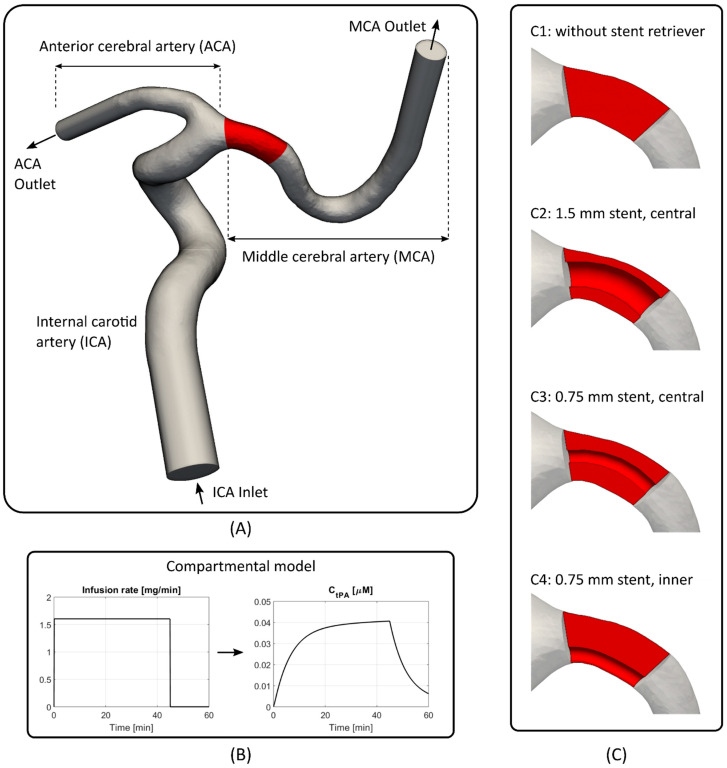
Overview of the computational model. (**A**) The reconstructed patient-specific arterial geometry consists of the internal carotid artery (ICA), anterior cerebral artery (ACA), and middle cerebral artery (MCA), with the thrombus shown in red. (**B**) The two-compartmental model provides concentrations of free phase proteins at the model inlet based on a given therapeutic dose. (**C**) Thrombi with different stent retriever configurations (C1–C4).

The 3D patient-specific geometry was reconstructed from 3D rotational angiography images using Materialise Mimics (v20.0, Materialise, Leuven, Belgium). The geometry consists of the internal carotid artery (ICA) which bifurcates into the anterior (ACA) and middle (MCA) cerebral artery branches (Figure 1A). Clot volumes were artificially implanted into the MCA branch, with the clot front located at the bifurcation between the ACA and MCA, as shown in red in Figure 1A. All simulated clot lengths are 5 mm along the centreline, and clot volumes (21.2–29.9 mm^3^) are within the range reported in a clinical study of M1 and M2 occlusions (mean thrombus volume of 129.0 ± 120.1, n = 214) [11]. This modelled clot size is at the lower end of reported ranges, and was selected for realistic simulation times. The frontal and rear faces of the clot are perpendicular to the centreline. Clots are modelled as a porous medium consisting of fibrin fibres, whereby clot density is controlled by altering fibrin fibre radius. A fibrin fibre radius of 65 nm was selected to be representative of medium density clots, and is the median of reported fine and coarse clots [9].

In this study, clots with four different stent retriever configurations were simulated by varying stent diameter and stent placement, as shown in Figure 1C. C1–C4 correspond to the various clot and stent configurations where: C1 is a clot without stent retriever; C2 with a 1.5 mm diameter stent retriever located along the centreline; C3 with a 0.75 mm stent retriever located along the centreline; and C4 with a 0.75 mm stent retriever located off the centre and close to the inner arterial wall. Stent retriever geometric details were not explicitly included, but their effects were modelled by removing clot volumes in place of the stent retriever, essentially creating a free passage through the clot with a free-fluid-thrombus interface. Unstructured meshes were created in Ansys ICEM (v17.0, ANSYS Inc., Canonsburg, PA, USA), and the various stent retriever configurations consisted of 1.5–1.7 million cells, which was sufficient for mesh independence of complete lysis times.

### 2.2. Computational Model Overview

Our developed computational model of thrombolysis [9,10] is composed of three coupled elements: blood flow; transport of key plasma proteins; and clot dissolution via fibrinolytic reaction kinetics. Blood flow is solved using the Navier–Stokes equations with an additional momentum source term which accounts for the resistance imposed by the clot. Transported proteins included in the model are plasminogen (PLG), plasmin (PLS) anti-plasmin (AP), and the infused thrombolytic drug: tissue plasminogen activator (tPA). Free phase protein transport in the blood is modelled using a series of convection-diffusion-reaction equations. Thrombus dissolution is based on the simultaneous binding and unbinding of fibrinolytic proteins with fibrin fibres in the thrombus, and the fibrinolytic reaction kinetics between the bound phase species proteins, acting to breakdown the fibrin fibres. Full details on the numerical model can be found in our previous work [9]. The numerical model for thrombolysis was successfully applied to MCA clots in patient-specific geometries [9,10], and results corroborated with the literature.

### 2.3. Numerical Details

A steady flow rate of 4.31 mL/s was prescribed at the ICA model inlet, which is the averaged ICA flow rate over a cardiac cycle [12]. This assumption was previously investigated using steady and pulsatile inlet flow rates in an idealised geometry, and it was found that lysis times and flow patterns were insensitive to flow pulsatility [9]. An in-house two-compartmental model [13] implemented in MATLAB was used to provide physiological and time-varying inlet boundary conditions for the four proteins: tPA; PLG; PLS; and AP. The model considers pharmacokinetic and pharmacodynamic (PKPD) effects of fibrinolytic proteins in plasma following intravenous administration of tPA—accounting for systemic and physiological processes. A standard therapeutic dose of 0.9 mg/kg of tPA [14] was administered as continuous infusion over 45 min. The two-compartmental model provides species concentrations at the computational inlet, at each time-step, as required in the free phase protein transport model. The infusion rate and corresponding concentration of free tPA, as calculated in the two-compartmental model, are shown in Figure 1B. Full details on the two-compartmental model can be found in [13].

Blood pressure is accounted for via a three-element Windkessel model [15] which was imposed at the ACA and MCA branch outlets, providing a physiological pressure boundary condition. Blood flow was assumed laminar, incompressible, and Newtonian, and vessel walls were assumed rigid with a no-slip boundary condition. The complete model equations, as described in Piebalgs et al. [9], are implemented in the open-source finite-volume software: OpenFOAM 4.0. All simulations were performed on 144 cores using the Cirrus UK National Tier-2 HPC Service at EPCC, and took between 3 and 12 days. Results were post-processed using ParaView and MATLAB.

## 3. Results

**Figure 2:**
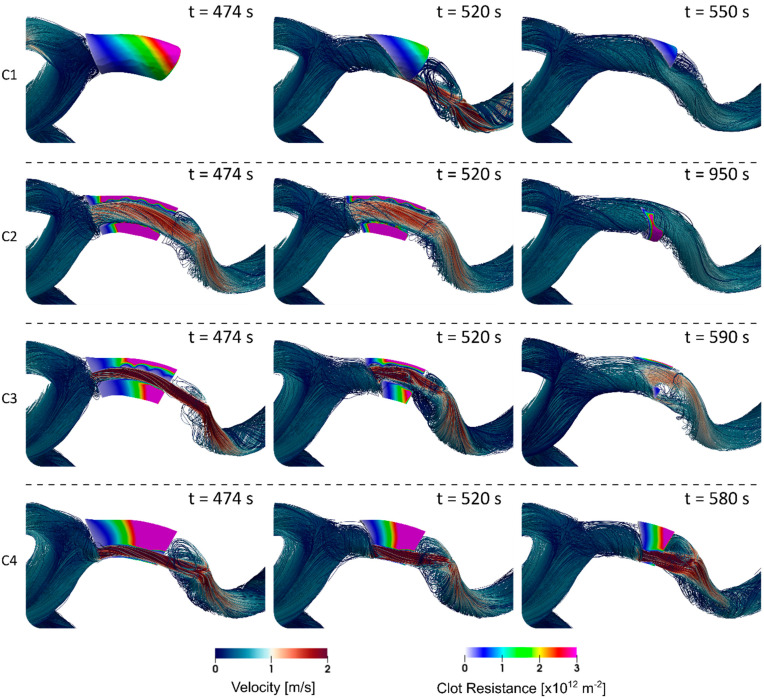
Flow and clot lysis behaviour over time, for (C1–C4). Streamlines are coloured by velocity magnitude, and clot volumes are coloured by clot resistance. t = 474 s is the last measured time before a decrease in clot volume is observed.

**Figure 3:**
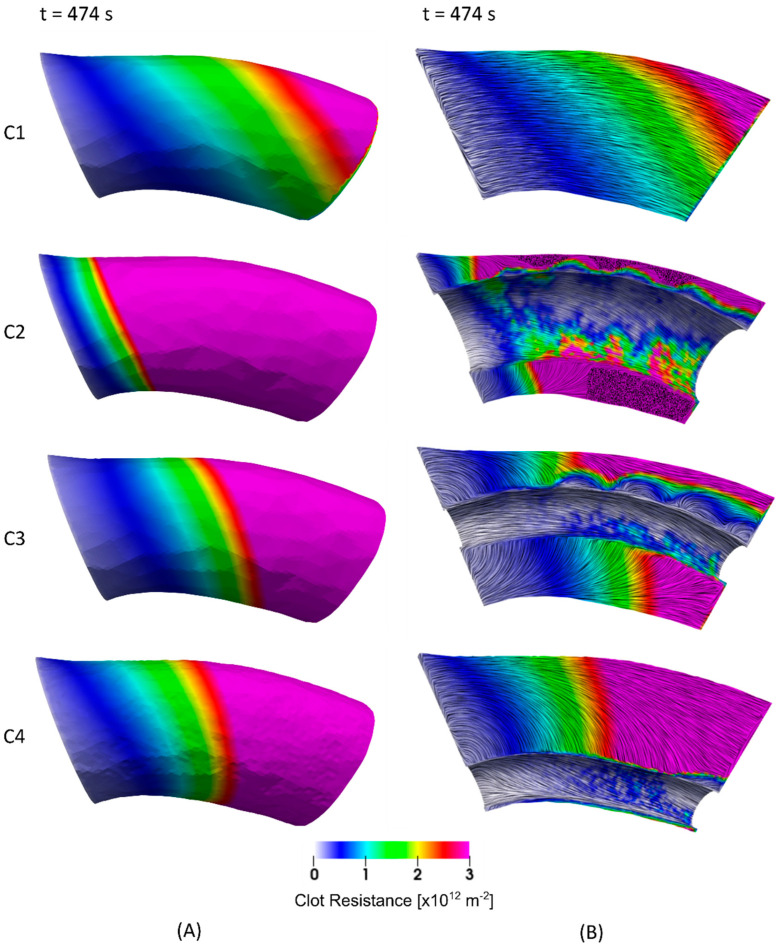
Clot volumes for C1 to C4, coloured by clot resistance at t = 474 s. (**A**) Shows the whole clot, and (**B**) shows the clot clipped to visualise internal patterns. Vector fields within the clots (**B**) are visualised using line integral convolution (LIC) projections.

**Figure 4:**
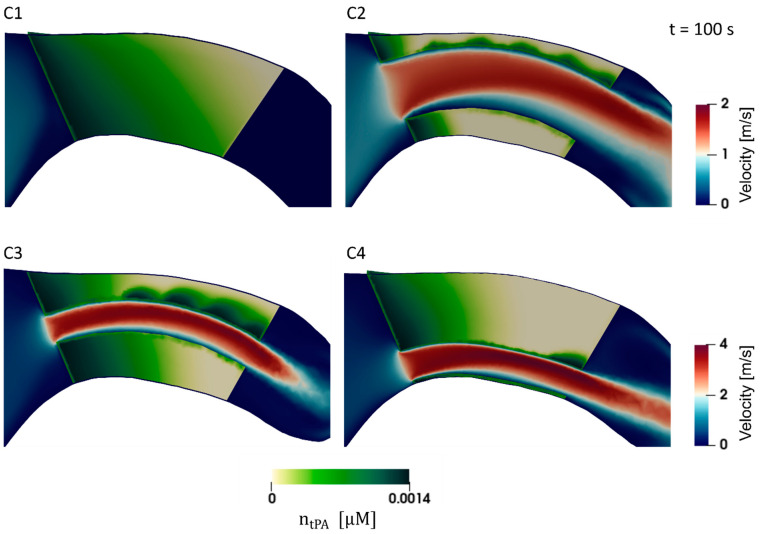
Clot volumes for C1 to C4, coloured by bound tPA (*n_tPA_*) at t = 100 s. Fluid regions through the MCA and clot are coloured by velocity magnitude; note the different scales on C1–C4.

**Figure 5:**
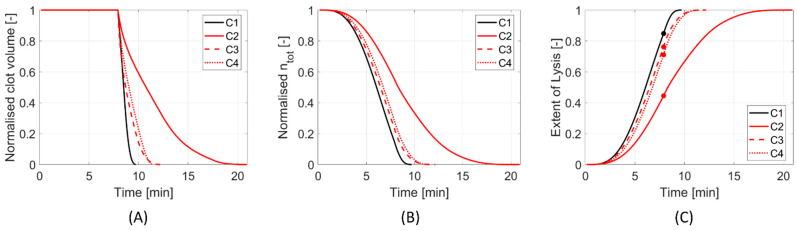
(**A**) Normalised clot volume, (**B**) normalised concentration of binding sites, and (**C**) extent of lysis, with t = 474 s highlighted with markers.

**Figure 6:**
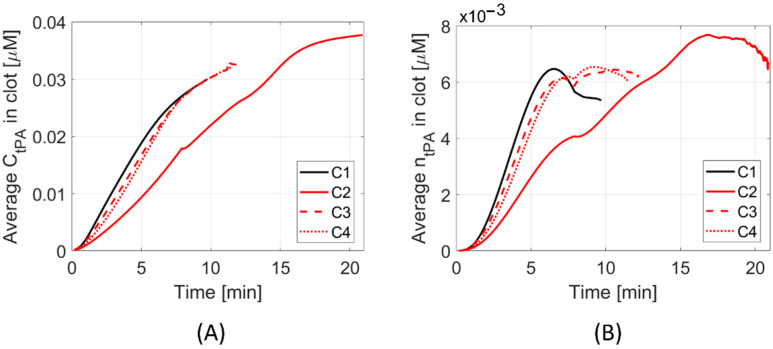
Volume-averaged concentrations of (**A**) free tPA and (**B**) bound tPA within the clot.

**Figure 7:**
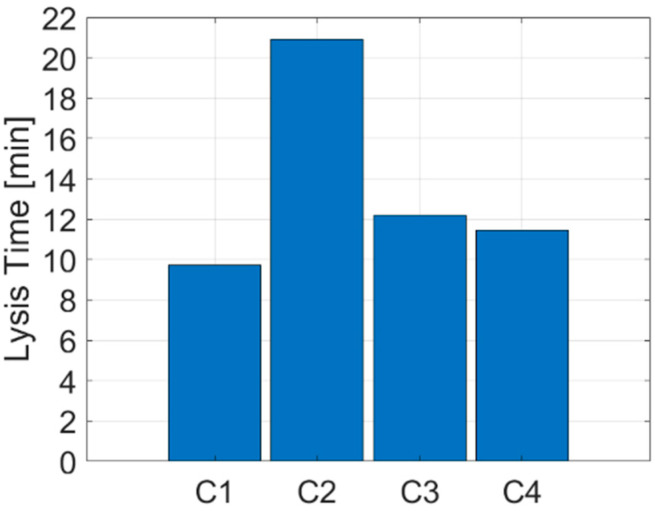
Complete recanalisation times for each configuration (C1–C4).

Results are presented for the four different clot and stent retriever configurations (C1–C4). Figure 2 shows C1–C4 at various times throughout clot dissolution. Flow patterns are visualised using velocity streamlines, and clot lysis behaviour is visualised with clot volumes coloured by clot resistance. Figure 3 compares clot resistance and blood flow patterns through the clot. Figure 4 shows bound tPA patterns within the clot, as well as velocity contours of the blood flow. Figure 5 shows temporal behaviours within each configuration, including normalised clot volume, normalised concentration of binding sites, and extent of lysis. Figure 6 shows volume-averaged concentrations of free tPA and bound tPA within the clots, and complete recanalisation times are shown in Figure 7. Fastest total recanalisation is achieved in the absence of a stent retriever (C1) in 9.7 min, followed by the smaller stent diameters (C3 and C4), which achieve recanalisation soon after by 12.2 and 11.5 min, respectively. The largest stent diameter (C2) achieves complete recanalisation significantly later, at 20.9 min.

For each stent retriever configuration, different lysis patterns are observed: 

C1. Clot lysis starts from the clot front and lyses uniformly over time, as can be visualised by clot resistance patterns in Figure 2. Lysis patterns are similar to those observed in our previous studies [9,10]. Partial recanalisation is achieved at 514 s along the inner curvature, producing a high velocity jet (Figure 2: C1, t = 520 s), after which, additional mixing occurs behind the clot at the rear face, however, lysis at the rear face is minimal. From the start of clot dissolution (475 s), it takes 108 s (1.8 min) for complete clot dissolution, and C1 lyses fastest of all configurations.

C2. Lysis starts from the clot front, as well as internally from the stent retriever channel, lysing radially towards the outer curvature arterial wall (Figure 2: C2, t = 474 s). The clot volume along the outer curvature breaks down first, leaving a fragment along the inner curvature, which is last to completely lyse (Figure 2: C2, t = 950 s). From the start of clot dissolution, it takes 780 s (13 min) for complete clot dissolution, and is the slowest of all configurations. 

C3. Similar to C2, lysis starts from the clot front, as well as radially from the stent retriever channel (Figure 2: C3, t = 474 s). The rate of lysis from the clot front is faster than radial lysis from the fluid-thrombus interface, and at t = 580 s, a region of the clot volume towards the inner curvature is lysed, providing a secondary channel helping to restore blood flow to the distal arteries (Figure 2: C3, t = 590 s). From the start of clot dissolution, it takes 256 s (4.3 min) for complete clot dissolution. 

C4. The thrombus lyses primarily from the clot front, and there is little radial dissolution from the stent retriever channel. Clot lysis patterns over time are similar to C1. From the start of thrombus dissolution, it takes 213 s (3.6 min) for complete clot dissolution.

## 4. Discussion

We have applied a previously validated model of thrombolysis to assess the impact of an idealised channel through an MCA thrombus (representing a deployed retrievable stent) on lysis. In this section, we discuss the physiology of clot lysis, and results are clinically evaluated.

### 4.1. Clot Lysis

There are two mechanisms for flow-enhanced thrombus lysis: (1) axial permeation (primary mechanism) and (2) radial permeation (secondary mechanism). Thrombus lysis is dependent on the various proteins binding with fibrin fibres. Plasmin cannot break down the fibrin fibres until it is bound—therefore, to maximise the rate of dissolution, as much binding as possible should be facilitated. An occluding thrombus (C1) creates a high-pressure gradient across the thrombus, forcing blood flow to permeate through the thrombus, which favours high rates of binding, whereas creating a channel through the thrombus (C2–C4) creates a path of lower resistance, allowing some tPA-carrying fluid to pass through the stent retriever channel, with less tPA-carrying fluid being ‘forced’ through the permeable thrombus. In the latter case, tPA convected through the stent retriever channel is unable to bind, unless it is in direct contact with the thrombus. Therefore, a significant amount of the drug passes through the channel, completely unused. These behaviours are observed in Figure 3 and Figure 4, where velocity vectors (Figure 3B) indicate fluid pathways through the clot, and correlate with higher localised regions of bound tPA (Figure 4).

Axial permeation: In C1, the fluid travels uniformly from front to back, whereas in C2–C4, the fluid that enters the clot front travels toward the stent retriever channel and re-joins the free-flowing blood flow through the channel (Figure 3B). In C2, blood flow entering the clot front re-joins the freestream in the shortest axial distance, followed by C3 and C4, respectively. These regions of axial permeation correlate with increased regions of binding and subsequent lysis (Figure 3B and Figure 4).

Radial permeation: Some thrombus lysis also occurs in the radial direction, as encountered in C2–C4. Radial permeation occurs in the stent retriever channel at the fluid-thrombus interface, predominantly along the outer curvature. Velocity contours (Figure 4) show that flow passing through the stent retriever channel is attached along the upper surface, but detaches from the lower surface in all stent retriever configurations. This relationship between velocity and radial permeation is shown in Figure 4. The high energy, attached flow along the upper surface facilitates higher rates of binding at the surface, and is even able to partially penetrate the clot radially. The flow that enters the clot then rapidly loses its energy and re-enters the fluid channel, owing to lower resistance (rather than continuing axially through the clot). Figure 4 clearly shows that more tPA binds along the upper surface where flow is attached, compared to the lower surface where flow separates, and velocities are small. Along the inner curvature, lower near wall energies result in lower concentrations of tPA.

The observed combined-mechanisms of clot lysis are further supported quantitatively by considering volume-averaged concentrations of free and bound tPA in the thrombi (Figure 6). More tPA passes through, and binds to the fibrin fibres in C1, followed closely by C3 and C4, which have smaller stent retriever idealised channels. In C2, there are much lower concentrations of free tPA passing through the thrombus and, therefore, lower rates of binding.

The plots in Figure 5 show that C3 and C4 exhibit similar lysis behaviours. C4 has a slower rate of dissolution than C3 up until ~11 min, then C4 suddenly breaks down faster. Towards the end of clot lysis, C3 only has a thin mural clot remaining along the outer curvature (parallel to flow direction), whereas C4 has a more substantial clot fragment remaining, with a clot front perpendicular to the fluid direction (Figure 2: C3 and C4, final times). At this stage in their respective lysing, C3 relies on radial permeation, whereas C4 benefits from axial permeation, which is observed to be the dominant mechanism, even though the remaining clot volume of C4 is larger than C3.

### 4.2. Clinical Evaluation

Previous groups using computational and in vitro thrombolysis models [16,17] have observed that the effect of tPA occurs mostly from axial permeation from the clot front rather than radial permeation at all available fluid-thrombus surfaces. Annand et al. [17] also observed a decrease in lysis rate after thrombus breakthrough when the pressure gradient driving axial permeation decreased, leaving radial permeation as the dominant lysis mechanism. These findings correlate well with our observations. An in vitro study of IVT in non-occlusive clots [18] observed trends of dissolution comparable to our simulations. Although the study also accounted for mechanical forces exerted by the blood flow on the clot, they concluded that higher velocity flows led to faster dissolution, in comparison to slower flows. We observed the same trend with our smaller and larger diameter stent retrievers, respectively.

Recent meta-analyses of thrombectomy trials found combined MT-IVT treatment compared favourably to MT alone in terms of functional outcomes, mortality, and successful recanalisation rates [2,3]. When MT was used in combination with thrombolysis, a reduced number of passes was noted compared to MT alone [2,19]. Two recent randomised control trials from Chinese centres (DIRECT-MT [5] and DEVT [6]) suggested non-inferiority of MT alone versus combined MT-IVT, however, wide non-inferiority margins were used, and it is unclear how applicable these results are without further studies. Conversely, data from the SKIP [7] randomised trial failed to show non-inferiority of MT alone. Subsequently, a meta-analysis of randomised clinical trials—including MR CLEAN-NO IV [4], and the aforementioned trials DIRECT-MT, DEVT, and SKIP—found that MT alone is non-inferior to IVT-MT based on functional independence at 90 days [8]. Despite the numerous recent meta-analyses, it is still unclear which treatment is preferential. The results of our pilot study suggest that if there is synergistic effect between MT and IVT, this is not from increased thrombus lysis whilst the stent is deployed. In clinical practice, the stent is retrieved very shortly after deployment, therefore, the lack of facilitation between thrombolysis and stent placement suggested by our results is unlikely to be clinically significant. The benefit of tPA effects may lie in recanalising foci of arterial occlusion distal to the main large vessel occlusion.

### 4.3. Limitations and Future Work

This is the first study to model combined IVT-MT treatment, which is a challenging multi-physics problem. Simulations in the present study took 3 to 12 days on high performance computing facilities. Owing to the complexities involved in modelling thrombolysis in the presence of a stent retriever, model simplifications were necessary for achievable computational times. These are discussed thoroughly below, along with suggestions for future work.

#### 4.3.1. Thrombolysis Model and Clot Properties

The complete process of thrombolysis spans multiple scales and includes blood flow, fibrin fibre dissolution, and cellular element transport and interactions (e.g., platelets). Our model is based on blood flow and fibrinolysis, meaning cellular component effects are neglected. Our previous work utilised the same model of thrombolysis in patient-specific arterial models of acute ischaemic stroke [9,10], and results agreed well with the literature. In the clot without a stent retriever, recanalisation times were within the ranges reported from clinical observation of MCA and basilar artery occlusions (23 ± 16 min) [20]. Another clinical observation of 73 patients with MCA occlusions reported time to beginning of recanalisation at 26 ± 18 min [21], and in this study, start of recanalisation occurred at ~9 min. Nonetheless, inclusion of cellular components could further improve model accuracy. Thrombosis can occur concurrently with mechanical thrombectomy procedures, owing to regions of stasis (e.g., at the clot front). In the numerical model of thrombolysis, thrombosis could be included, although, this will significantly increase computational costs. A relatively small clot volume was used in this study. A clinical observation into IVT in MCA occlusions found that partial recanalisation was unsuccessful in clot lengths > 8 mm [22]. The interaction between IVT-MT in larger clots would need to be modelled to understand the relationship in these settings.

#### 4.3.2. Mechanical Thrombectomy

To reduce computational complexity, an idealised cylindrical channel was created through the thrombus, and stent retriever geometric details were not explicitly included. In reality, the channel calibre is irregular, and the stent struts imprint/penetrate the thrombus when deployed, likely creating areas of recirculation and stasis, as well as increasing the available binding surface area. Inclusion of stent retriever geometries would require significant localised mesh refinement, and increasing simulation times, and was omitted for this reason. Future work could include detailed stent retriever geometry.

In this study, the thrombus volume was reduced by the volume of the stent retriever channel, which is an approximation, given that no thrombus is removed by the stent until this is retrieved. Instead, the thrombus is displaced and/or compressed by stent in situ. Despite smaller initial volumes of thrombus in C2–C4 than the configuration without a stent retriever (C1), these still took longer overall to completely lyse. Thrombus composition was assumed to be homogeneous in this study, but in reality, thrombi are heterogeneous [23], which would likely alter lysis patterns. Future work could address this via a precursor simulation of stent deployment, similar to [24,25] in heterogeneous thrombi.

#### 4.3.3. Translation to Practice

The long term aims of this work are to create a modelling tool of clinical relevance which can inform decision making in the treatment of acute ischaemic stroke. In the present pilot study, various simplifications were necessary for achievable simulation times, and in future work, these simplifications can be sensitivity tested. The model will be used to vary and evaluate numerous parameters, such as: clot volume, location, and composition; different stent retriever devices and deployment scenarios; and parameters relating to thrombolytic therapy, such as drug dosage and different thrombolytic agents.

## 5. Conclusions

We conducted simulations of combined IVT-MT treatment of various idealised stent retriever configurations in an MCA thrombus to evaluate combined treatment performance in terms of complete recanalisation times and lysis patterns. Four stent-thrombus configurations were simulated by varying stent diameter and location. With a channel in situ, the thrombus was slower to lyse, and this phenomenon was accentuated when increasing the diameter of the channel. The thrombus without a stent retriever achieved fastest complete recanalisation in 9.7 min. The two smaller stent retriever configurations (0.75 mm diameters located along the centreline and off the centre, close to the inner arterial wall) achieved similar recanalisation times of 12.2 and 11.5 min, respectively. The largest stent diameter (1.5 mm, located along the centreline) achieved complete recanalisation significantly later than other configurations, at 20.9 min.

Two mechanisms of clot lysis were established: axial permeation and radial permeation. The presence of a stent retriever channel allowed for radial permeation to occur at the fluid-thrombus interface, however, our results show that axial permeation was the primary mechanism of clot lysis. Permeation in the radial direction was much slower compared to the axial direction because of weaker secondary velocities. As a result, binding primarily occurs local to the fluid-thrombus interface normal to the direction of flow.

The findings of this study provide a first step in understanding the complex relationship between IVT and MT, and provide a basis for future studies in improving the efficacy of these combined treatments.
